# Effects of Arch Support Pad Stiffness on Lower-Limb Biomechanics During Single-Leg Landing

**DOI:** 10.3390/sports13090323

**Published:** 2025-09-11

**Authors:** Chu-Hao Li, Qiu-Qiong Shi, Kit-Lun Yick, Ming-Yu Hu, Shi-Wei Mo

**Affiliations:** 1 School of Fashion and Textiles, The Hong Kong Polytechnic University, Hong Kong SAR 999077, China; chu-hao.li@connect.polyu.hk (C.-H.L.); tcyick@polyu.edu.hk (K.-L.Y.); mingyu.hu@connect.polyu.hk (M.-Y.H.); 2 Research Institute for Sports Science and Technology, Hong Kong SAR 999077, China; 3 Laboratory of Human Kinesiology & Performance, School of Physical Education, Shenzhen University, Shenzhen 518060, China; moshiwei@szu.edu.cn

**Keywords:** arch support, arch pad, single-leg landing, landing biomechanics

## Abstract

Arch structure is a crucial interface between the human body and the ground during landing tasks, but the biomechanical effects of arch support stiffness remain insufficiently explored. This study examines the effects of arch supports with different stiffnesses on lower-limb biomechanics during landing. Twelve male participants (six normal arches, six flat feet) performed a single-leg drop landing from a 45 cm height under four arch support conditions: no arch support pad (NAP), soft-stiffness arch support pad (SAP), medium-stiffness arch support pad (MAP), and high-stiffness arch support pad (HAP). Dominant lower-limb joint angles and moments in the sagittal plane and vertical ground reaction force (vGRF)-related parameters—time to peak vGRF, peak vGRF, and max loading rate—were recorded using a motion capture system and force plate. Data were analyzed using one-way repeated measures analysis of variance (ANOVA). Arch pad stiffness significantly affected ankle and knee kinematics. The NAP condition exhibited significantly higher ankle plantarflexion at initial contact (*p* ≤ 0.01), as well as larger range of motion (ROM) of the knee (*p* = 0.03) and hip (*p* < 0.01), compared to the use of a SAP or MAP. The use of a HAP resulted in a significantly lower peak ankle dorsiflexion moment and larger peak knee flexion angle than the other conditions (*p* ≤ 0.04). The peak knee extension moment was the highest when using a NAP, and was significantly higher than that shown with the use of a MAP or HAP (*p* ≤ 0.02). No significant differences were observed in hip joint moments or vGRF-related parameters across conditions (*p* ≥ 0.52). These results indicate that hard-stiffness arch support pads modulate lower-limb mechanics during landing, potentially enhancing shock absorption and reducing knee loading.

## 1. Introduction

Landing is a fundamental movement pattern frequently encountered in athletic training, particularly in sports involving jumping, deceleration and cutting movements such as basketball, volleyball and badminton. During landings, athletes may experience ranging from 2 to 7 times body weight (BW) [1], with even higher peaks possible in abrupt sport-specific maneuvers. The lower extremities sequentially absorb and transmit these forces from the foot to the ankle, knee, and hip. Consequently, poor force distribution during this kinetic chain of the lower extremities can lead to excessive joint loading [2], which explains why high impact landings are greatly associated with musculoskeletal injuries, including ligament injuries, tendinopathy, joint pain, and arthritis [3,4]. Importantly, interventions such as arch supports can alter plantar mechanics, redistribute forces along the kinetic chain, and thereby influence lower-limb loading patterns [5]. These biomechanical effects highlight their potential role in both optimizing performance and preventing landing-related injuries.

To reduce the impact forces and risk of injury during landings, “cushioning” technologies have been incorporated into the sports surface [6] and footwear because they help reduce the landing impact [7] and pain [8], improve dynamic balance [9] and enhance athletic performance [10]. The arch, a critical weight-bearing and shock-absorbing component of the foot, influences the lower-extremity biomechanics by regulating plantar pressure distribution and impact absorption. Zhang et al. [11] suggested that muscle fiber contraction and musculoskeletal coordination help to dissipate landing impact. Arch support insoles provide mechanical support to the medial arch, which promotes neutral foot and knee alignment and alters the joint kinematics, thereby influencing lower-limb biomechanics and potentially preventing injury [12]. For instance, medial longitudinal arch support pads increase the maximum knee flexion angle during landing compared to unsupported conditions [13]. However, other landing biomechanical studies showed that arch support orthotics do not significantly affect the sagittal and coronal plane angles of the knee and ankle joints during landing [14]. The contradicting results may be attributed to differences in drop height, arch support material and whether the dominant or non-dominant leg was tested. Lam et al. [9] investigated the right leg by using a polyurethane arch support, although the landing height was not reported. In contrast, Takata et al. [13] studied the non-dominant leg during landings from a platform at a height of 30 cm, but the material of the arch support was not reported. Thus, the relationship between arch support and injury prevention remains unclear. Furthermore, load distribution and postural control are highly relevant to clinical populations [15], particularly individuals with neurological disorders such as stroke, who often exhibit impaired proprioception, reduced weight transfer between legs, and altered anticipatory postural adjustments. Interventions such as arch support insoles and targeted exercise programs had been shown to influence gait parameters and lower-limb joint kinematics, potentially improving functional mobility and safety during daily activities [16]. Single-leg landing provides a complementary perspective by revealing acute lower-limb adaptations to sudden, high-impact loads, complementing habitual gait analyses to offer a comprehensive understanding of how arch support interventions affect biomechanics, performance and injury risk.

A successful landing requires sufficient muscle strength and joint stability to prevent lower-limb injuries. Arch support insoles are frequently prescribed for musculoskeletal pain and biomechanically driven injuries. Previous research suggests that insole stiffness may potentially modify the mechanical input into the musculoskeletal system during landing [17]. However, findings on this topic remain inconsistent. For example, the second peak of the vertical ground reaction force (vGRF) was higher when wearing hard-stiffness shoes than medium- and soft-stiffness shoes [18]. Conversely, Nolan [19] found that changes in midsole density did not significantly affect the landing impact or loading rate during landing from spike jumps among female volleyball players. These discrepancies may stem from the ability of the athletes to adapt via compensatory strategies—that is, to compensate for changes in the midsole cushioning during landing, such as altering landing patterns or modifying joint kinematics [20,21]. From a biomechanical perspective, increased midsole bending stiffness has been shown to reduce arch deformation and alter the behavior of plantar muscle–tendon units, potentially impacting energy storage, shock attenuation, and lower-limb biomechanics [22]. Similarly, arch support insoles can influence lower-limb kinematics; for instance, functional arch support insoles increased ankle dorsiflexion, but reduced knee and hip flexion during walking and jogging [23]. Controlling plantar arch motion with targeted insoles can modulate lower-limb biomechanics by redistributing plantar pressure and reducing excessive arch collapse. This adjustment influences ankle alignment and knee loading, enhancing energy absorption and lowering abnormal joint stress. Consequently, insoles may improve landing performance efficiency and reduce the risk of injury during landing. Nevertheless, the specific effects of targeted arch regional stiffness, as opposed to general insole or midsole hardness, remain underexplored, highlighting the need for systematic investigation into how regional stiffness characteristics affect landing mechanics.

Given the prevalence of landing-related injuries in athletic activities and the inconsistent results of previous studies, further investigation is warranted. While most prior studies have focused on general insole stiffness, the biomechanical effects of targeted arch support pad stiffness remain largely unexplored. Therefore, this study aimed to examine how arch support pads with different stiffness levels affect lower-limb kinematics and kinetics during single-leg landing. We hypothesized that a stiffer arch support pad would increase knee flexion angle during landing and reduce peak vGRF.

## 2. Materials and Methods

### 2.1. Participants

The sample size was determined beforehand using G*Power (version 3.1.9.7) software. Based on a repeated-measures analysis of variance ANOVA with a large effect size (f = 0.8), α = 0.05, and power = 0.8, a minimum of 10 participants was required. Twelve healthy male subjects were recruited (age: 26.8 ± 2.3 years; height: 1.72 ± 0.05 m; body mass: 67.0 ± 9.1 kg; BMI: 22.7 ± 2.5 kg/m^2^). Six participants had normal arches and six had flat feet, which were determined using the arch index method from footprints [24]. All subjects were actively engaged in lower-limb-dominant sports such as basketball, running, and badminton, with an average training frequency of 3–5 sessions per week for the past 2 years. The recruitment criteria included the absence of lower-extremity surgery, no lower-extremity injuries or pain reported in the previous 6 months, no medical problems (e.g., osteoarthritis, diabetes, and neurological disorders), and no history of orthotic use or evident foot deformities that might affect performance. All of the subjects signed an informed consent form after the study briefing. The study was approved by the ethics committee of The Hong Kong Polytechnic University Ethics Committee (HSEARS20240520004) in accordance with the Declaration of Helsinki.

### 2.2. Arch Support Pads

**Figure 1:**
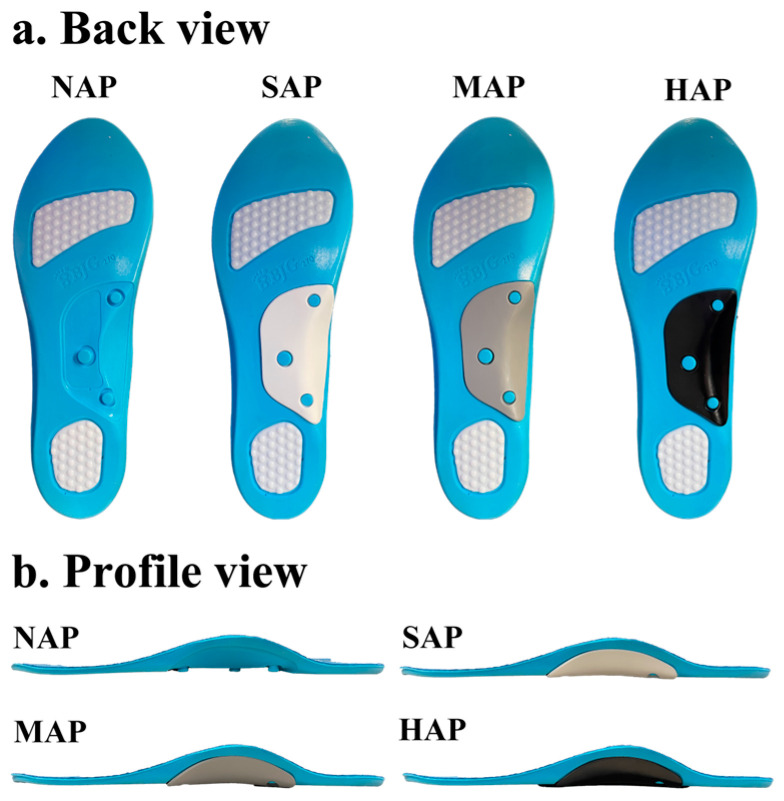
Experimental arch support pad conditions. NAP: no arch support pad; SAP: soft-stiffness arch support pad; MAP: medium-stiffness arch support pad; HAP: high-stiffness arch support pad.

Four polyurethane (PU) arch support pads were tested: no arch support pad (NAP), soft-stiffness arch support pad (SAP), medium-stiffness arch support pad (MAP), and high-stiffness arch support pad (HAP). The stiffness of each pad was measured using a durometer hardness tester (GS-719N, Type A, Teclock Co., Nagano, Japan) by applying vertical pressure to the central flat area of the pad; measurements were repeated five times and averaged [25]. The selected stiffness values (NAP: 24.8 ± 1.6 Shore A; SAP: 36.0 ± 2.7 Shore A; MAP: 56.4 ± 3.6 Shore A; HAP: 78.8 ± 3.6 Shore A) were chosen to represent the typical stiffness range of commercially available arch support insoles, from soft to high stiffness, as reported in clinical footwear design research [26,27]. Each pad measured 86 mm in length, 46 mm in width, and 17 mm in height, and was positioned under the medial longitudinal arch region of the insole. The anatomical placement was standardized across participants according to insole size rather than individual foot morphology. All insoles included an arch support pad and had uniform specifications (forefoot thickness: 4.5 mm; arch height: 25.0 mm; rearfoot thickness: 6.3 mm), ensuring reproducibility (Figure 1).

### 2.3. Experimental Protocol

A single-blinded randomized crossover design was employed to evaluate the effects of four different arch support pads (NAP, SAP, MAP, and HAP) on lower-limb kinematics and kinetics during single-leg landing. Randomization was achieved using a computer-generated random sequence of four conditions (www.randomizer.org, accessed on 22 March 2025) prior to testing, and allocation was performed by an independent researcher not involved in data collection to ensure blinding.

The experiment was carried out in the Human Performance Laboratory of the university. The height and body mass of the subjects were measured with a calibrated scale and stadiometer. A motion capture system (Vicon Metrics Ltd., Oxford, UK) with nine cameras was used to capture the motion trajectory, and the kinetic data were obtained by using a force plate (AMTI, Watertown, MA, USA). Vicon and Nexus 2.15.0 software was used to synchronously collect and process kinematics and kinetic data, and the sampling frequency of kinematics and kinetic was established at 100 and 1000 Hz, respectively. All participants were required to wear standardized sportswear (i.e., sports shorts and shoes). The footwear used a traditional training shoe (Little White, Qingdao Doublestar Ltd., Qingdao, China) characterized by a rubber outsole, a heel-to-toe drop of 0.4 mm, a stack height of 18.2 mm, and without additional arch support or protective features. This model was selected due to its popularity among recreational players in China.

**Figure 2:**
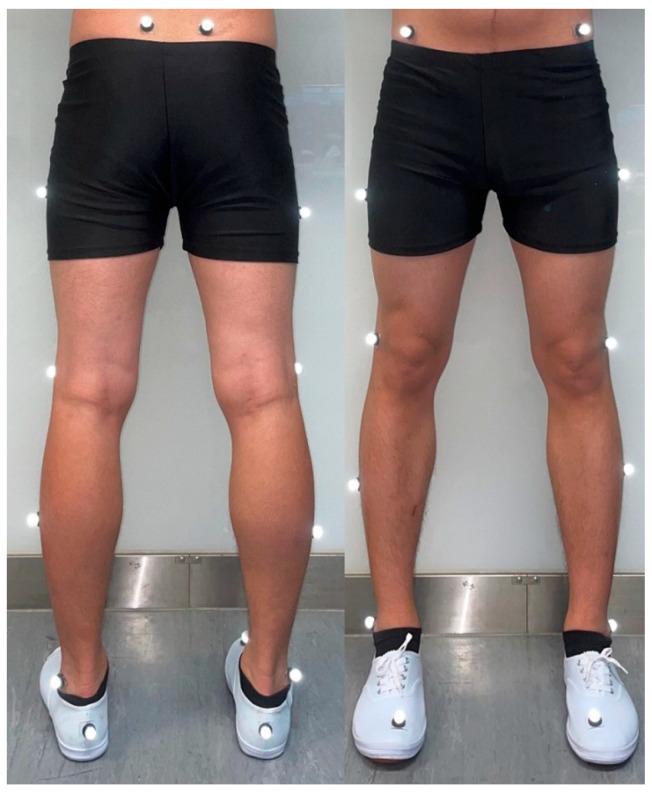
Marker set with sixteen retro-reflective markers.

Prior to data collection, participants completed a dynamic warm-up protocol, including high knee pulls, Frankenstein’s, and forward gate swings [28]. The warm-up exercises were performed twice for a distance of 15 m each, followed by comprehensive muscle stretching. Sixteen retro-reflective markers (Figure 2) with 14 mm dimensions were affixed to the participants’ skin using double-sided tape by a same experienced researcher, markers were positioned on both lower limbs according to the Vicon Clinical Manager protocol over the anterior superior iliac spine, posterior superior iliac spine, lateral midthigh, lateral femoral condyle, lateral midcalf, lateral malleolus, posterior calcaneus, and head of the second metatarsal [29].

The participants were instructed to stand on the platform and perform a drop landing protocol by stepping off the platform with a single leg [30]. All participants were right-leg dominant. The dominant leg (the preferred leg in daily exercise, typically better suited for landing or kicking a ball) was used to land on the force plate, as non-contact injuries are more commonly observed in the dominant leg of male athletes [31]. A trial was considered valid if the participant stepped off the platform with arms crossed over the chest and landed stably with the dominant leg, maintaining balance for at least 3 s. Additionally, all participants were acclimated to the experimental environment and practiced single-leg drop landings from a 45 cm height platform to ensure proper execution. Familiarization was deemed sufficient when participants successfully completed at least three consecutive landings with correct technique. Between each trial, participants were allowed at least 30 s rest interval with approximately 5 min of rest between the four conditions to ensure sufficient recovery and prevent fatigue. Finally, a total of 12 valid trials (three per condition) were collected from each participant, and three trials within each condition were averaged to generate a representative dataset.

### 2.4. Data Collection and Processing

The raw data of the reflective markers and GRF were processed using Vicon Nexus software (version 2.15.0) for static calibration and further analysis. Firstly, the trajectories were labeled, trimmed, and checked for breaks due to marker occlusion, with missing data interpolated and visually checked for accuracy. Then, a fourth-order zero-lag Butterworth digital filter at cut-off frequencies of 6 Hz to smooth and filter trajectory data [32,33]. The extracted anthropometric parameters obtained from the static position were subsequently applied to the dynamic data. Finally, export the processed data in CSV format for each valid trial. All of the data exported from Vicon Nexus were then imported into MATLAB R2021b (MathWorks, Natick, Massachusetts, USA) for further processing. The data were further analyzed in MATLAB using custom-written scripts, which automated the extraction of biomechanical parameters to minimize manual error. For all trials, the initial contact was defined as the point where vertical GRF exceeded 20 N, and the landing phase was defined as the period from initial contact point to maximum knee flexion [34]. Positive values were defined as hip and knee flexions and ankle dorsiflexion in the sagittal plane. Negative values were defined as hip and knee extensions and ankle plantarflexion following the *Plug-in Gait Reference Guide* [35].

### 2.5. Statistical Analysis

All data are reported as the mean ± standard deviation (SD). For significant variables, mean difference (MD) and a 95% confidence interval (CI) are provided to facilitate the interpretation of clinical significance. All statistical analyses were performed by using SPSS Statistics (version 26.0; IBM Corp., Armonk, NY, USA). Each participant completed all four arch support pad conditions (NAP, SAP, MAP, HAP), and outcome measures were collected under each condition. Because the same participants were measured across all conditions, a one-way repeated-measures ANOVA was used to account for within-subject variability.

When significant main effects were identified, Bonferroni post hoc analyses were conducted to determine the differences between conditions. Confidence intervals for statistical analyses were set at 95%, and the alpha level was set at 0.05. The effect size (ES) was calculated by using partial eta-squared (*η*^2^) for the ANOVA, and was interpreted as small (0.01 ≤ *η*^2^ < 0.06), medium (0.06 ≤ *η*^2^ < 0.14), or large (*η*^2^ ≥ 0.14); the ES (Cohen’s d) was used to assess *t*-tests, interpreted as small (<0.5), medium (0.5–0.8), and large (>0.8) [36].

## 3. Results

### 3.1. Kinematics

Table 1 shows that the arch support pad had no main effect on the knee (F = 1.59, *p* = 0.23, *η*^2^ = 0.13) and hip (F = 0.24, *p* = 0.84, *η*^2^ = 0.02) joint angles at initial contact, nor for the ankle (F = 1.92, *p* = 0.17, *η*^2^ = 0.15) and hip (F = 1.55, *p* = 0.23, *η*^2^ = 0.12) joint angles at maximum knee flexion. However, significant main effects with the use of the different arch support pad insoles were observed for the ankle joint angle at initial contact (F = 5.60, *p* = 0.01, *η*^2^ = 0.34) and knee joint angle at maximum knee flexion (F = 3.67, *p* < 0.05, *η*^2^ = 0.25). Post hoc tests with Bonferroni’s correction revealed that the NAP group exhibited a significantly higher ankle plantarflexion angle at initial contact than the MAP (MD  =  −2.61, 95% CI from −4.43 to −0.80 degrees, *p* < 0.01, ES = 0.60), and HAP (MD  =  3.13, 95% CI from −5.62 to −0.64 degrees, *p* = 0.01, ES = 0.69) groups. Additionally, the HAP group showed significantly larger knee flexion angle at maximum knee flexion when compared to the SAP (MD  =  2.33, 95% CI from 0.15 to 4.52 degrees, *p* = 0.04, ES = 0.25) and MAP (MD  =  4.06, 95% CI from 0.63 to 7.49 degrees, *p* = 0.03, ES = 0.46) groups. Although no significant main effects from the arch support pads were observed for the ROM of the ankle during landing (F = 3.13, *p* = 0.06, *η*^2^ = 0.22), significant differences were found in the ROM of the knee (F = 3.80, *p* = 0.04, *η*^2^ = 0.20) and hip (F = 4.61, *p* = 0.01, *η*^2^ = 0.30) joints across the four conditions. A post hoc analysis revealed that the NAP group showed a significantly larger ROM of the knee than the MAP group (MD  =  1.78, 95% CI from 0.13 to 3.44 degrees, *p* = 0.03, ES = 0.30), while the NAP group showed a significantly larger ROM of the hip compared to the MAP group (MD  =  1.94, 95% CI from 0.67 to 3.22 degrees, *p* < 0.01, ES = 0.60).

### 3.2. Kinetics

Table 2 lists the kinetic outcomes in the sagittal plane. The use of the arch support pad insoles with varying stiffness did not significantly affect the time to peak vGRF (F = 0.44, *p* = 0.70, *η*^2^ = 0.04), peak vGRF (F = 0.39, *p* = 0.75, *η*^2^ = 0.04), or maximum loading rate (F = 0.05, *p* = 0.99, *η*^2^ = 0.01). Similarly, no significant main effects from the arch support pad insoles can be observed for the peak knee flexion moment (F = 0.85, *p* = 0.47, *η*^2^ = 0.07), or peak hip flexion moment (F = 1.24, *p* = 0.31, *η*^2^ = 0.10) across the four conditions. However, arch support pads significantly affect the peak ankle dorsiflexion moment (F = 3.70, *p* = 0.04, *η*^2^ = 0.25) and peak knee extension moment (F = 5.27, *p* = 0.01, *η*^2^ = 0.32). A post hoc analysis revealed that the HAP group exhibited a significantly lower peak ankle dorsiflexion moment than the NAP (MD  =  −0.24, 95% CI from −0.44 to −0.05 Nm/kg, *p* = 0.02, ES = 1.72), SAP (MD  =  −0.15, 95% CI from −0.26 to −0.04 Nm/kg, *p* = 0.01, ES = 1.16), and MAP (MD  =  −0.15, 95% CI from −0.28 to −0.02 Nm/kg, *p* = 0.01, ES = 1.23) groups. Furthermore, a significantly higher peak knee extension moment can be observed in the NAP group compared to the MAP (MD  =  −0.24, 95% CI from −0.44 to −0.03 Nm/kg, *p* = 0.02, ES = 0.44), and HAP (MD  =  −0.28, 95% CI from −0.53 to −0.04 Nm/kg, *p* = 0.02, ES = 0.53) groups. In contrast, arch support pads neither significantly affect the peak ankle plantarflexion moment (F = 0.59, *p* = 0.57, *η*^2^ = 0.05) nor the peak hip extension moment (F = 0.75, *p* = 0.52, *η*^2^ = 0.06) among the four conditions.

## 4. Discussion

This study found that arch support pad stiffness primarily influenced ankle and knee mechanics during single-leg landing, while the hip joint remained relatively unaffected. Stiffer pads altered ankle plantarflexion at initial contact and increased knee flexion at landing, while the absence of arch support was associated with greater overall joint excursion at both the knee and hip joints. These findings highlight the ankle as the critical joint in early landing control and demonstrate how changes in medial arch stiffness can cascade proximally to influence knee joint′s landing strategies.

### 4.1. Kinematics

At initial contact, the ankle joint angle was significantly affected by the arch support pads, while MAP and HAP conditions reduced ankle plantarflexion compared to the NAP group. This finding suggests that the appropriate arch support pad can accommodate the position of the ankle while landing by offering more structural support to the medial longitudinal arch and enhancing foot stability. This finding aligns with previous research by Davidson et al. [37], who reported that arch support orthoses provide support to the medial midfoot, thereby improving postural control and receptor sensory fields on the plantar surface. In contrast, no significant differences were found in the knee or hip joint angles at initial contact across four conditions, indicating that early landing strategies are primarily influenced by the distal joints. As the ankle is the first part of the foot to interact with the ground, it must rapidly adapt to the surface conditions and body orientation. This early adjustment facilitates alignment of the lower-limb segments, helps to maintain balance, and prevents excessive impact transfer.

However, Lam et al. [14] found that arch support insoles did not alter ankle plantarflexion angle at initial contact; this discrepancy may not only be attributed to the use of softer PU materials in their study, which preserved plantar sensation, but also to use of shoes. For instance, Lam et al. focused on basketball footwear, where the ability to enhance ankle stability stands as one of the most crucial characteristics. The use of basketball shoes may counteract the impact of arch height on the ankle joint during landing. Moreover, participant characteristics (trained basketball players vs. active amateurs) may have further contributed to the divergent outcomes. In contrast, the present study employed arch supports with varying stiffness, allowing for more pronounced alterations in ankle joint response during landing. These results emphasize the critical role of ankle mechanics in initial landing control and highlight the potential of appropriately stiff arch support pads to facilitate safer, more efficient movement strategies during high-impact activities such as jump training.

At the moment of maximum knee flexion, participants who used a HAP had the largest knee flexion angle among four conditions, which was significantly higher than that shown with the use of a SAP or MAP. This increased flexion was consistent with prior studies, suggesting that greater knee flexion serves as a shock attenuation strategy during landing [38], which may reduce the risk of musculoskeletal injuries during high-impact movements [11]. Additionally, significant differences can be observed in the ROM of knee (*p* = 0.04) and hip (*p* = 0.01), with the NAP group exhibited significantly larger ROM as opposed to the MAP group. This finding may indicate that in the absence of medial arch support, greater proximal joint excursion is required to maintain postural stability and absorb impact forces. From a kinetic chain perspective, this increased ROM may reflect compensatory strategies for force generation, transmission, and control across segments [39]. Such compensatory strategies, while beneficial in the short term for maintaining stability, have been associated with elevated joint loading and a higher risk of overuse injuries, particularly at the knee and hip [40,41].

### 4.2. Kinetics

In the present study, no significant differences were observed in vGRF-related variables (time to peak vGRF, peak vGRF, and maximum loading rate) among four conditions. This finding suggest that the magnitude of landing impact at the whole-body level was not altered by the stiffness of arch support pads. However, it is worth noting that vGRF alone may not fully reflect the potential protective or risk-modifying effects of arch supports. Previous studies demonstrated that foot orthoses can alter plantar pressure distribution [42,43,44,45,46], thereby influencing local loading patterns, even in the absence of detectable changes in vGRF. It is therefore possible that the arch support pads in this study redistributed forces across the plantar surface without substantially affecting the global vertical force outcome. Future studies should incorporate plantar pressure distribution to capture these localized effects.

Despite the lack of vGRF differences, significant alterations were observed in joint moments. Specifically, the HAP reduced the peak ankle dorsiflexion moment compared to the NAP, SAP, and MAP conditions. This finding suggests that stiffer arch support pads may restrict excessive dorsiflexion demand at the ankle joint, potentially by enhancing the structural support of the medial longitudinal arch. Reduced ankle dorsiflexion moments could lower the mechanical demand on the ankle extensors during landing, thereby improving joint stability. This interpretation aligns with previous research showing that foot orthotic can modify ankle kinetics and redistribute loading patterns during running [47]. These changes in joint moments may be partially explained by alterations in muscle activation patterns and joint coupling. Enhanced medial arch support likely modulates the activation timing and magnitude of key stabilizing muscles such as the tibialis anterior, gastrocnemius, and quadriceps [48]. By redistributing muscle activity, excessive dorsiflexion and knee extension demands can be reduced, improving stability. Moreover, stiffer arch supports may influence intersegmental coordination along the kinetic chain. By limiting midfoot dorsiflexion, ankle–knee–hip coupling and muscle recruitment patterns become more efficient [49,50], allowing for proximal joints to adopt safer postures during high-impact landings.

The absence of differences in hip moments underscores that the biomechanical effects of arch support were localized to the ankle and knee joints [51], which are directly involved in absorbing ground reaction forces during landing. The hip joint, as a proximal and inherently stable structure, plays a dominant role in maintaining trunk and pelvic control, particularly under high-impact conditions such as single-leg landings [39]. Since the primary function of the hips is to absorb and distribute forces to maintain balance, subtle changes in arch support stiffness may not substantially alter their biomechanical role.

To our knowledge, there were no published reports defining a minimal clinically important difference (MCID) for lower-joint biomechanics measured during a single-leg landing task. Therefore, it is not possible to compare our observed differences (ankle dorsiflexion moment: from −0.24 to −0.15 Nm/kg; knee extension moment: from −0.24 to −0.28 Nm/kg) against established thresholds. Nonetheless, given the ES from 0.44 to 1.72, these changes are unlikely due to measurement error alone, and are, therefore, likely biomechanically and clinical meaningful. Future studies should incorporate test–retest reliability designs under the same equipment settings to establish normative MCID values for these variables during single leg landing. Meanwhile, although this study did not examine the effects of long-term use, it has been shown that the use of foot orthotics may lead to adaptive changes. For instance, a four-month application of arch support has been shown to modify lower-limb coordination during gait in children with flexible flatfoot [50].

### 4.3. Practical Applications

This study provides guidance for footwear design, injury prevention, and performance optimization in landing-related activities. Arch support pads, particularly with appropriate level of stiffness, help regulate ankle positioning at initial contact and reduce excessive plantarflexion. Stiffer supports may benefit individuals with ankle instability or knee valgus by enhancing intersegmental coordination and distributing impact loads more effectively. Moreover, the observed effects on ankle and knee moments suggest that arch supports can help prevent overuse injuries such as patellofemoral pain and Achilles tendinopathy. Athletes engaged in repetitive jump–landing tasks may therefore integrate appropriate arch support devices into footwear which may reduce excessive muscle activation and energy expenditure required to stabilize the lower limb, effectively lowering the mechanical cost of landing. Finally, as effectiveness may vary with foot morphology and neuromuscular control, individualized assessment and fitting are recommended to maximize benefits.

### 4.4. Limitations

Several limitations should be acknowledged. First, this study focused on acute responses to different arch support stiffness during single-leg landing; thus, the chronic adaptations to prolonged use remain unknown. Second, as all participants were male, the findings may not be generalizable to females, who exhibit differences in foot morphology, neuromuscular control, and footwear preferences. Third, the arch support orthotics used in this study were not customized to individual foot types (e.g., high-arched or low-arched) or athlete-specific needs [2]. The orthotic contours and fit can affect both wear comfort and biomechanical performance during landing. Fourth, this study tested landings from a single height, which limits the validity for sports requiring variable landing intensities. Fifth, potential placebo effects cannot be ruled out because participants were not blinded to the intervention. Finally, the use of controlled single-leg drop landings may not fully capture the demands of sport-specific tasks, such as multidirectional jumps or fatigue-induced landings.

Future studies should therefore investigate both acute and chronic responses across diverse populations (e.g., female athletes, different foot morphologies), explore the influence of multiple landing tasks and sport-specific conditions, and adopt designs that address placebo or expectation effects. Moreover, determining MCID for lower-limb kinematics and kinetics during landing would enhance the translational value of arch support interventions for injury prevention and performance optimization.

## 5. Conclusions

Arch support pad stiffness significantly modulates ankle and knee biomechanics during single-leg landing, with stiffer arch supports reducing ankle plantarflexion and knee extension moments while increasing knee flexion, suggesting enhanced shock absorption and joint protection. Hip moments and vGRF were unaffected, highlighting the distal-specific effects of arch supports. These findings provide guidance for footwear design and injury prevention, underscoring the potential for individualized arch support interventions to optimize performance in athletic populations.

## Figures and Tables

**Table 1:** Mean ± SD for joint angles in sagittal plane (unit: degrees).

Variable	NAP	SAP	MAP	HAP	*p*	*η* ^2^
Initial contact						
Ankle	−24.65 ± 4.20	−21.71 ± 5.74	−22.04 ± 4.51	−21.52 ± 4.86	0.01	0.34
Knee	−0.78 ± 3.84	0.20 ± 4.76	−0.29 ± 4.51	0.51 ± 4.46	0.23	0.13
Hip	10.86 ± 5.68	10.58 ± 4.70	11.05 ± 5.38	11.46 ± 4.98	0.84	0.02
Max knee flexion						
Ankle	25.86 ± 4.44	25.83 ± 4.59	24.92 ± 5.36	26.87 ± 4.90	0.168	0.15
Knee	31.97 ± 8.09	32.40 ± 8.97	30.68 ± 8.08	34.73 ± 9.66	<0.05	0.25
Hip	27.08 ± 5.92	25.52 ± 6.88	25.46 ± 6.12	27.57 ± 6.89	0.231	0.12
Range of motion						
Ankle	51.07 ± 6.65	48.06 ± 6.10	47.58 ± 6.31	48.90 ± 7.59	0.056	0.22
Knee	32.75 ± 5.86	32.20 ± 6.80	30.97 ± 5.84	34.23 ± 7.01	0.04	0.26
Hip	17.51 ± 3.62	16.49 ± 4.20	15.57 ± 2.85	17.39 ± 3.70	0.01	0.30

Note. NAP: no arch support pad; SAP: soft-stiffness arch support pad; MAP: medium-stiffness arch support pad; HAP: high-stiffness arch support pad.

**Table 2:** Mean ± SD for kinetics outcomes in sagittal plane.

Variable	NAP	SAP	MAP	HAP	*p*	*η* ^2^
Vertical GRF (BW)						
Time to Peak vGRF (ms)	0.06 ± 0.01	0.06 ± 0.01	0.06 ± 0.01	0.06 ± 0.01	0.70	0.04
Peak vGRF (BW)	4.04 ± 0.45	3.95 ± 0.36	3.99 ± 0.36	3.99 ± 0.40	0.75	0.04
Max loading rate (BW/s)	66.33 ± 11.89	66.30 ± 11.47	65.55 ± 10.17	66.25 ± 9.77	0.99	0.01
Peak joint moment (Nm/kg)						
Ankle dorsiflexion	3.42 ± 0.53	3.33 ± 0.47	3.33 ± 0.39	3.18 ± 0.44	0.04	0.25
Knee flexion	1.36 ± 0.62	1.36 ± 0.69	1.29 ± 0.63	1.41 ± 0.56	0.47	0.07
Hip flexion	4.94 ± 1.77	4.61 ± 1.74	4.40± 1.47	4.49 ± 1.47	0.31	0.10
Ankle plantarflexion	−0.18 ± 0.09	−0.19 ± 0.08	−0.18 ± 0.07	−0.19 ± 0.08	0.57	0.06
Knee extension	−1.71 ± 0.57	−1.55 ± 0.61	−1.48 ± 0.51	−1.43 ± 0.51	0.01	0.32
Hip extension	−3.33 ± 2.08	−3.64 ± 2.75	−3.23 ± 2.16	−3.45 ± 2.25	0.52	0.06

Note. NAP: no arch support pad; SAP: soft-stiffness arch support pad; MAP: medium-stiffness arch support pad; HAP: high-stiffness arch support pad.

## Data Availability

The data that support the findings of this study are available from the corresponding author upon reasonable request. The data are not publicly available due to privacy and ethical restrictions.

## Abbreviations

The following abbreviations are used in this manuscript:

BW	Body weight
CI	Confidence intervals
ES	Effect size
HAP	High-stiffness arch-support pad
MD	Mean difference
MCID	Minimal clinically important difference
MAP	Medium-stiffness arch-support pad
NAP	No arch-support pad
PU	Polyurethane
ROM	Range of motion
SAP	Soft-stiffness arch-support pad
SD	Standard deviation
vGRF	Vertical ground reaction force

## References

[1] Mizrahi J., Susak Z. (1982). Analysis of Parameters Affecting Impact Force Attenuation during Landing in Human Vertical Free Fall. Eng. Med..

[2] Powell D.W., Hanson N.J., Long B., Williams D.S.B.I. (2012). Frontal Plane Landing Mechanics in High-Arched Compared with Low-Arched Female Athletes. Clin. J. Sport Med..

[3] Decker M.J., Torry M.R., Wyland D.J., Sterett W.I., Richard Steadman J. (2003). Gender differences in lower extremity kinematics, kinetics and energy absorption during landing. Clin. Biomech..

[4] Radin E.L., Martin R.B., Burr D.B., Caterson B., Boyd R.D., Goodwin C. (1984). Effects of mechanical loading on the tissues of the rabbit knee. J. Orthop. Res..

[5] Chen H., Zhang Q., Biró I. Effects of Arch Support Pads and Insoles on Gait Parameters and Plantar Mechanics During Running in Adults with Flatfoot. Proceedings of the 2024 IEEE 7th International Conference and Workshop Óbuda on Electrical and Power Engineering (CANDO-EPE).

[6] Hovey S., Henry W., Judge L.W., Avedesian J.M., Dickin D.C. (2021). The effect of landing type on kinematics and kinetics during single-leg landings. Sports Biomech..

[7] Wang L., Hong Y., Li J.-X., Zhou J.-H. (2012). Comparison of Plantar Loads During Running on Different Overground Surfaces. Res. Sports Med..

[8] Banwell H.A., Mackintosh S., Thewlis D. (2014). Foot orthoses for adults with flexible pes planus. a systematic review. J. Foot Ankle Res..

[9] Lam W.-K., Lee W.C.-C., Ng S.-O., Zheng Y. (2019). Effects of foot orthoses on dynamic balance and basketball free-throw accuracy before and after physical fatigue. J. Biomech..

[10] Mills K., Blanch P., Chapman A.R., McPoil T.G., Vicenzino B. (2010). Foot orthoses and gait. a systematic review and meta-analysis of literature pertaining to potential mechanisms. Br. J. Sports Med..

[11] Zhang S.N., Bates B.T., Dufek J.S. (2000). Contributions of lower extremity joints to energy dissipation during landings. Med. Sci. Sports Exerc..

[12] Shi Q.Q., Li P.L., Yick K.-L., Li N.-W., Jiao J. (2022). Effects of contoured insoles with different materials on plantar pressure offloading in diabetic elderly during gait. Sci. Rep..

[13] Takata Y., Sugimoto M., Iwamoto K., Kitsunai I., Sugiyama K., Kimura K. (2020). Medial longitudinal arch pad influences landing control of the lower limbs during single-leg jump-landing. Health.

[14] Lam W.-K., Cheung C.C., Zhiguan H., Leung A.K. (2022). Effects of shoe collar height and arch-support orthosis on joint stability and loading during landing. Res. Sports Med..

[15] Liu Y.-T., Tsai H.-T., Hsu C.-Y., Lin Y.-N. (2021). Effects of orthopedic insoles on postural balance in patients with chronic stroke. A randomized crossover study. Gait Posture.

[16] Vecchio M., Chiaramonte R., Alessandro D., Buccheri E., Finocchiaro P., Scaturro D., Mauro G.L., Cioni M. (2024). Do proprioceptive training strategies with dual-task exercises positively influence gait parameters in chronic stroke? A systematic review. J. Rehabil. Med..

[17] Fu W., Liu Y., Zhang S. (2013). Effects of footwear on impact forces and soft tissue vibrations during drop jumps and unanticipated drop landings. Int. J. Sports Med..

[18] Zhang S., Clowers K., Kohstall C., Yu Y.-J. (2005). Effects of Various Midsole Densities of Basketball Shoes on Impact Attenuation during Landing Activities. J. Appl. Biomech..

[19] Nolan K.J. (2004). The Influence of Variations in Shoe Midsole Density on the Impact Force and Kinematics of Landing in Female Volleyball Players. Doctoral Dissertation.

[20] De Wit B., De Clercq D., Aerts P. (2000). Biomechanical analysis of the stance phase during barefoot and shod running. J. Biomech..

[21] Hamill J., Russell E.M., Gruber A.H., Miller R. (2011). Impact characteristics in shod and barefoot running. Footwear Sci..

[22] Cigoja S., Asmussen M.J., Firminger C.R., Fletcher J.R., Edwards W.B., Nigg B.M. (2020). The Effects of Increased Midsole Bending Stiffness of Sport Shoes on Muscle-Tendon Unit Shortening and Shortening Velocity. a Randomised Crossover Trial in Recreational Male Runners. Sports Med.-Open.

[23] Zhao X., Wang M., Fekete G., Baker J.S., Wiltshire H., Gu Y. (2021). Analyzing the effect of an arch support functional insole on walking and jogging in young, healthy females. Technol. Health Care.

[24] Cavanagh P.R., Rodgers M.M. (1987). The arch index. a useful measure from footprints. J. Biomech..

[25] Healy A., Dave D., Chockalingam N. (2011). Effect of insole material on plantar pressure. Footwear Sci..

[26] Anderson J., Williams A.E., Nester C. (2020). Development and evaluation of a dual density insole for people standing for long periods of time at work. J. Foot Ankle Res..

[27] Su S., Mo Z., Guo J., Fan Y. (2017). The effect of arch height and material hardness of personalized insole on correction and tissues of flatfoot. J. Healthc. Eng..

[28] LaPorta J.W., Brown L.E., Coburn J.W., Galpin A.J., Tufano J.J., Cazas V.L., Tan J.G. (2013). Effects of Different Footwear on Vertical Jump and Landing Parameters. J. Strength Cond. Res..

[29] Russell K.A., Palmieri R.M., Zinder S.M., Ingersoll C.D. (2006). Sex differences in valgus knee angle during a single-leg drop jump. J. Athl. Train..

[30] Shimokochi Y., Ambegaonkar J.P., Meyer E.G., Lee S.Y., Shultz S.J. (2013). Changing sagittal plane body position during single-leg landings influences the risk of non-contact anterior cruciate ligament injury. Knee Surg. Sports Traumatol. Arthrosc..

[31] Brophy R., Silvers H.J., Gonzales T., Mandelbaum B.R. (2010). Gender influences: The role of leg dominance in ACL injury among soccer players. Br. J. Sports Med..

[32] Winter D.A. (2009). Biomechanics and Motor Control of Human Movement.

[33] Dadfar M., Sheikhhoseini R., Jafarian M., Esmaeili A. (2021). Lower extremity kinematic coupling during single and double leg landing and gait in female junior athletes with dynamic knee valgus. BMC Sports Sci. Med. Rehabil..

[34] Richardson M.C., Chesterton P., Taylor A., Evans W. (2024). The effect of surface on knee landing mechanics and muscle activity during a single-leg landing task in recreationally active females. Phys. Ther. Sport.

[35] Plug-in Gait Reference Guide. https://help.vicon.com/download/attachments/11378719/Plug-in%20Gait%20Reference%20Guide.pdf.

[36] Cohen J. (2013). Statistical Power Analysis for the Behavioral Sciences.

[37] Davidson D.M., Werd M.B., Knight E.L. (2010). Prefabricated Insoles and Modifications in Sports Medicine. Athletic Footwear and Orthoses in Sports Medicine.

[38] Wikstrom E.A., Powers M.E., Tillman M.D. (2004). Dynamic stabilization time after isokinetic and functional fatigue. J. Athl. Train..

[39] Mendiguchia J., Ford K.R., Quatman C.E., Alentorn-Geli E., Hewett T.E. (2011). Sex Differences in Proximal Control of the Knee Joint. Sports Med..

[40] Heiderscheit B.C. (2010). Lower extremity injuries. is it just about hip strength?. J. Orthop. Sports Phys. Ther..

[41] Weiss K., Whatman C. (2015). Biomechanics Associated with Patellofemoral Pain and ACL Injuries in Sports. Sports Med..

[42] Desmyttere G., Leteneur S., Hajizadeh M., Bleau J., Begon M. (2020). Effect of 3D printed foot orthoses stiffness and design on foot kinematics and plantar pressures in healthy people. Gait Posture.

[43] Shimokochi Y., Yong Lee S., Shultz S.J., Schmitz R.J. (2009). The relationships among sagittal-plane lower extremity moments. implications for landing strategy in anterior cruciate ligament injury prevention. J. Athl. Train..

[44] Yu B., Garrett W.E. (2007). Mechanisms of non-contact ACL injuries. Br. J. Sports Med..

[45] Cheng J., Zeng Q., Lai J., Zhang X. (2022). Effects of arch support doses on the center of pressure and pressure distribution of running using statistical parametric mapping. Front. Bioeng. Biotechnol..

[46] Nigg B.M., Nurse M.A., Stefanyshyn D.J. (1999). Shoe inserts and orthotics for sport and physical activities. Med. Sci. Sports Exerc..

[47] Mündermann A., Nigg B.M., Neil Humble R., Stefanyshyn D.J. (2003). Foot orthotics affect lower extremity kinematics and kinetics during running. Clin. Biomech..

[48] Piri E., Sobhani V., Jafarnezhadgero A., Arabzadeh E., Shamsoddini A., Zago M., Granacher U. (2025). Effect of double- density foot orthoses on ground reaction forces and lower limb muscle activities during running in adults with and without pronated feet. BMC Sports Sci. Med. Rehabil..

[49] Dami A., Payen E., Farahpour N., Robb K., Isabelle P.-L., Moisan G. (2024). Medially wedged foot orthoses generate greater biomechanical effects than thin-flexible foot orthoses during a unilateral drop jump task on level and inclined surfaces. Clin. Biomech..

[50] Jafarnezhadgero A., Mousavi S.H., Madadi-Shad M., Hijmans J.M. (2020). Quantifying lower limb inter-joint coordination and coordination variability after four-month wearing arch support foot orthoses in children with flexible flat feet. Hum. Mov. Sci..

[51] Ferber R., Davis I.M., Williams D.S. (2005). Effect of foot orthotics on rearfoot and tibia joint coupling patterns and variability. J. Biomech..

